# Epidemiologic, Clinical, and Genetic Characteristics of Human Infections with Influenza A(H5N6) Viruses, China

**DOI:** 10.3201/eid2807.212482

**Published:** 2022-07

**Authors:** Wenfei Zhu, Xiyan Li, Jie Dong, Hong Bo, Jia Liu, Jiaying Yang, Ye Zhang, Hejiang Wei, Weijuan Huang, Xiang Zhao, Tao Chen, Jing Yang, Zi Li, Xiaoxu Zeng, Chao Li, Jing Tang, Li Xin, Rongbao Gao, Liqi Liu, Min Tan, Yuelong Shu, Lei Yang, Dayan Wang

**Affiliations:** National Institute for Viral Disease Control and Prevention, Chinese Center for Disease Control and Prevention, Beijing, China (W. Zhu, X. Li, J. Dong, H. Bo, J. Liu, Jiaying Yang, Y. Zhang, H. Wei, W. Huang, X. Zhao, T. Chen, Jing Yang, Z. Li, X. Zeng, C. Li. J. Tang, L. Xin, R. Gao, L. Liu, M. Tan, Y. Shu, L. Yang, D. Wang);; Chinese Center for Disease Control and Prevention, Beijing (C. Li);; World Health Organization Collaborating Centre for Reference and Research on Influenza, Beijing (W. Zhu, X. Li, J. Dong, H. Bo, J. Liu, Jiaying Yang, Y. Zhang, H. Wei, W. Huang, X. Zhao, T. Chen, Jing Yang, Z. Li, X. Zeng, J. Tang, L. Xin, R. Gao, L. Liu, M. Tan, Y. Shu, L. Yang, D. Wang);; Key Laboratory for Medical Virology, National Health and Family Planning Commission, Beijing (W. Zhu, X. Li, J. Dong, H. Bo, J. Liu, Jiaying Yang, Y. Zhang, H. Wei, W. Huang, X. Zhao, T. Chen, Jing Yang, Z. Li, X. Zeng, J. Tang, L. Xin, R. Gao, L. Liu, M. Tan, Y. Shu, L. Yang, D. Wang);; Sun Yat-sen University, Guangzhou, China (Jiaying Yang, Y. Shu)

**Keywords:** influenza, influenza A(H5N6) viruses, human infection, epidemiology, genetic characteristics, zoonoses, China, respiratory infections

## Abstract

The recent rise in the frequency of influenza A(H5N6) infections in China has raised serious concerns about whether the risk for human infection has increased. We surveyed epidemiologic, clinical, and genetic data of human infections with A(H5N6) viruses. Severe disease occurred in 93.8% of cases, and the fatality rate was 55.4%. Median patient age was 51 years. Most H5N6 hemagglutinin (HA) genes in human isolates in 2021 originated from subclade 2.3.4.4b; we estimated the time to most recent common ancestor as June 16, 2020. A total of 13 genotypes with HA genes from multiple subclades in clade 2.3.4.4 were identified in human isolates. Of note, 4 new genotypes detected in 2021 were the major causes of increased H5N6 virus infections. Mammalian-adapted mutations were found in HA and internal genes. Although we found no evidence of human-to-human transmission, continuous evolution of H5N6 viruses may increase the risk for human infections.

Various subtypes of avian influenza viruses (AIVs) circulate globally in wild birds. Domestic poultry are also susceptible to these viruses and, occasionally, AIVs have become able to cross the species barrier to infect humans (*1*–*4*). In China, situations in which human infections with AIVs have occurred can be complicated; many subtypes of AIVs have been reported, including influenza A(H5N1), A(H5N6), A(H7N4), A(H7N9), A(H9N2), A(H10N8), and A(H10N3) (*4*,*5*). Highly pathogenic avian influenza (HPAI) A(H5) viruses have continually caused worldwide outbreaks in both wild birds and poultry, with some spillover to humans, most notably 863 HPAI A(H5N1) cases, 456 of which were fatal (*6*).

In April 2014, the first human infection with HPAI A(H5N6) virus was reported in Sichuan Province, China (*1*). Since then, human cases have been continuously documented in China. By the end of 2021, 66 cases had been documented globally, 36 of which were fatal. Of those, 65 cases were in China, and the remaining case was reported in Laos.

Since its emergence in Guangdong Province in 1996, HPAI H5 type viruses became endemic in birds in China and other regions and developed into distinct clades (*7*). The clade 2.3.4.4 H5 viruses were first reported in migratory birds in eastern China in 2013, followed by outbreaks in both wild and domestic birds in South Korea at the beginning of 2014. Thereafter, clade 2.3.4.4 H5 viruses spread westward and eastward from Asia to other continents, accompanied by multiple reassortment events between clade 2.3.4.4 H5 viruses and other AIVs circulating in wild and domestic birds (*8*,*9*). Clade 2.3.4.4 H5N6 AIV has been mainly endemic among birds in China and southeast Asia since 2013 (*10*–*12*), gradually replacing H5N1 as a dominant AIV subtype in poultry across southern China (*13*). Similar to H7N9 AIV, reassortments among H5N6 and H9N2 AIVs occurred dynamically (*14*–*16*). Furthermore, many other subtypes of AIVs were documented to donate their internal genes to H5N6, including AIVs from wild birds (*11*,*17*,*18*). Thus, H5N6 genotypes diversified both in poultry and humans (*11*,*16*–*18*).

Genetic and biologic characteristics indicated that H5N6 viruses were highly pathogenic in chickens (*19*–*21*). Studies have shown that some H5N6 viruses of clade 2.3.4.4 possessed the ability to bind both avian-origin and human-origin sialic acid receptors and had the ability to attach to human tracheal epithelial and alveolar tissues (*22*). Although H5N6 viruses were not as pathogenic in mice and ferrets as their parental clade 2.3.4 H5N1 viruses, they exhibited greater transmissibility than H5N1 viruses in a ferret model (*22*,*23*). Thus, clade 2.3.4.4 H5N6 viruses demonstrated higher potential to transmit among humans.

During the COVID-19 pandemic, seasonal influenza infections notably decreased worldwide compared with previous flu seasons (*24*). However, human infections with zoonotic influenza viruses do not appear to have decreased. Of note, a recent rise in the frequency of H5N6 cases was observed (*25*). For the purposes of preparing for future possible pandemics, investigating the epidemiologic, clinical, and genetic characteristics of H5N6 AIVs that infect humans is critical.

## Methods

### Study Cases

In China, all laboratory-confirmed influenza A(H5N6) cases are reported through a national surveillance system. Patients with respiratory tract specimens that test positive for H5 and N6 by real-time reverse transcription PCR are confirmed as H5N6 infections. Demographic, epidemiologic, and basic clinical data on influenza A(H5N6) cases are collected on standardized forms. We included information regarding age; sex; place of residence; symptoms at illness onset; comorbidities associated with increased risk for influenza complications; dates of illness onset, hospital admission, death or discharge, and clinical treatments; and potential exposures to domestic or retail animals and visits to live poultry markets in our analysis.

### Ethics

The National Health Commission of the People’s Republic of China determined that the collection of data on each H5N6 case was part of a continuing public health investigation of an emerging outbreak. Thus, the ethics approval was exempt from official review by our institutional review board.

### Virus Isolation

We used original samples from human cases for H5/N6 subtyping by real-time reverse transcription PCR (*26*). We selected positive samples for virus isolation. We conducted the isolation of H5N6 viruses in a BioSafety Level 3 laboratory by inoculating 0.2 mL of original sample into 9- to 11-day specific pathogen–free embryonated chicken eggs. After samples incubated for 36–48 hours at 37°C, we harvested allantoic fluids.

### RNA Extraction and Genome Sequencing

We extracted RNA from the original samples and isolated viruses by using the QIAamp viral RNA mini kit (QIAGEN, https://www.qiagen.com) and performed sequencing as follows. As previously described (*27*), we subjected extracted RNA to reverse transcription and amplification. We then implemented whole-genome sequencing of FluA on the MiSeq or Miniseq high-throughput sequencing platform (Illumina, https://www.illumina.com) (*28*). We predominately conducted data analysis and genome sequence acquisition by using a pipeline established in our laboratory. We trimmed low-quality reads, sampled the filtered reads, and de novo assembled using Velvet version 1.2.10 (https://guix.gnu.org/en/packages/velvet-1.2.10) and Newbler Assembler version 2.5. We blasted contigs against a database generated by CD-HIT that clusters all FluA sequences collected from the GISAID EpiFlu database (http://www.gisaid.org) and National Center for Biotechnology Information (NCBI) Influenza Virus Database. We selected sequences with the highest similarity as references and used bowtie2 version 2.1.0 (https://sourceforge.net/projects/bowtie-bio/files/bowtie2/2.1.0) for read mapping. We obtained FluA genome sequences by extracting the consensus sequences from the mapping results, with a coverage depth of at least 30× at each site on the 8 segments. We submitted the genome sequences of influenza A(H5N6) viruses determined in this study to GISAID (Appendix Table 1).

### Sequence Alignment and Phylogenetic Analysis

We collected all H5N6 human isolate sequences, including sequences from GISAID, and downloaded the top 100 sequences of avian viruses with high similarity to each representative sequence through BLAST (https://blast.ncbi.nlm.nih.gov/Blast.cgi) from GISAID. We used CD-HIT to reduce the sequence redundancy of phylogenetic analysis and performed sequence alignments by using MAFFT software version 6.857b (https://mafft.cbrc.jp/alignment/software). We constructed a maximum-likelihood phylogenetic tree for the nucleotide sequences of each gene of selected influenza viruses under the GTRGAMMAR model with 1,000 bootstrap replicates, using RAxML (*29*). To estimate the time to most recent common ancestor (tMRCA) of hemagglutinin (HA) and neuraminidase (NA) genes of the H5N6 virus, we selected nonredundant subdatasets to run time-measured Bayesian Markov chain Monte Carlo analysis by BEAST v1.10.4 (*30*). We used the SRD06 substitution model (*31*) and the uncorrelated relaxed molecular clock model and set the Bayesian skygrid coalescent as the tree prior. We ran the Bayesian Markov chain Monte Carlo for up to 1 × 10^8^ steps, with samples for each 10,000 steps to achieve convergence. We used tracer version 1.6 (https://bioweb.pasteur.fr/packages/pack@Tracer@v1.6) to examine effective sample size values >200.

### Statistical Analysis

We summarized continuous variables as either means + SD or medians with interquartile ranges (IQRs). For categorical variables, we calculated the percentages of patients in each category. We used parametric tests to analyze normally distributed variables and nonparametric tests to analyze non–normally distributed variables. We used an unpaired Student *t* test, Wilcoxon rank-sum test, χ^2^ test, or Fisher exact test, as appropriate, to compare the epidemiologic and clinical characteristics of subgroups of patients who were infected with influenza A(H5N6) virus before or during 2021. We calculated 95% CIs for the means of normally distributed data and the risk estimates by using the binomial distribution. We considered a p value of <0.05 statistically significant. We performed all analyses in SAS version 9.4 (SAS Institute, Inc., https://www.sas.com).

## Results

### Epidemiologic Findings 

During April 21, 2014–December 31, 2021, a total of 65 cases of human influenza A(H5N6) infection were reported in China; illness onset dates occurred during April 13, 2014–December 31, 2021. The case-fatality rate (CFR) was 55.4% (36/65). Most cases were reported through pneumonia surveillance and were identified by Chinese National Influenza Surveillance Network laboratories; however, 15 cases in 2020 and 2021 were first identified through third-party sequencing agencies and reported to Chinese National Influenza Surveillance Network laboratories for confirmation.

**Figure 1:**
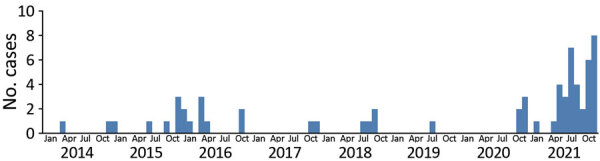
Temporal distribution of 65 human infections with influenza A(H5N6) virus, by month, China, April 21, 2014–December 31, 2021.

**Figure 2:**
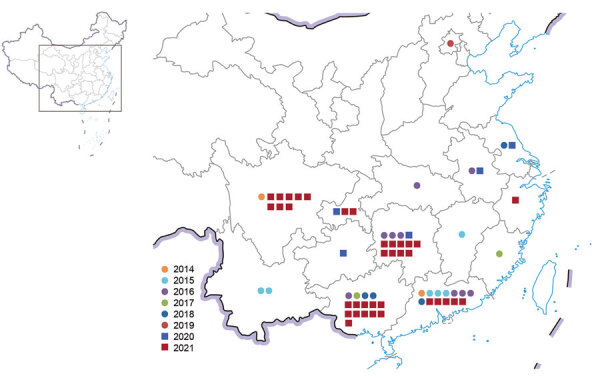
Spatial distribution of 65 human infections with influenza A(H5N6) virus, China, by year, April 21, 2014–December 31, 2021.

The number of human cases of H5N6 infection reported for each year from 2014 to 2021 was 2 for 2014, 6 for 2015, 9 for 2016, 2 for 2017, 4 for 2018, 1 for 2019, 5 for 2020, and 36 for 2021 (Figure 1). These cases were distributed across 14 provinces in China; most (64/65) cases were detected in southern China (Figure 2). The H5N6 cases were predominantly in adults; median patient age was 51 (IQR 36–57) years (Table 1). More than half (45/65, 69.2%) of H5N6 cases occurred in persons 18–59 years of age. Men accounted for 50.8% (33/65) of H5N6 infections (Table 1).

**Table 1:** Characteristics of 65 laboratory-confirmed cases of human infection with avian influenza A(H5N6) virus, China*

Characteristic	No. case-patients
Median age (IQR)	51 (36–57)
Age group, y	
0–17	8 (12.3)
18–59	45 (69.2)
>60	12 (18.5)
Sex	
M	33 (50.8)
F	32 (49.2)
Residence	
Urban	34 (52.3)
Rural	31 (47.7)
Fatalities (CFR)	36 (55.4)
Poultry exposure	
Any exposure to poultry	61 (93.8)
Visited live poultry market	38 (62.3)
Exposure to backyard poultry	28 (45.9)
Exposure to sick or dead poultry	23 (37.7)
Processed poultry	28 (45.9)
A(H5) positive in related bird or avian-related environment	55 (90.2)
Comorbidities†	31 (47.7)
Disease severity	
Mild	4 (6.2)
Severe‡	61 (93.8)
Treatment	
Oseltamivir	39 (60.0)
Mechanical ventilation	41 (63.1)
ECMO	14 (21.5)
Admission to ICU	51 (78.5)
Complications§	
Yes	56 (86.2)
No	7 (10.8)
Unknown	2 (3.1)

*Values are no. (%) except as indicated. CFR, case-fatality rate; ECMO, extracorporeal membrane oxygenation; ICU, intensive care unit; IQR, interquartile range. †Only comorbidities associated with a high risk for influenza complications (*32*,*33*) were counted here, including chronic respiratory disease, asthma, chronic cardiovascular disease, diabetes, chronic liver disease, chronic kidney disease, immunosuppressed status, and neuromuscular disorders. ‡Severe symptoms were defined according to the Diagnosis and Treatment Plan for Influenza issued by the National Health Commission of the People’s Republic of China (http://www.gov.cn/zhengce/zhengceku/2020-11/05/5557639/files/74899af960ff4f228e280d08b60d2af1.pdf). §Complications included secondary bacterial pneumonia, bronchitis, exacerbations of underlying respiratory conditions, laryngotracheobronchitis; and other less common complications may occur (*33*).

Poultry exposure is the main risk factor for human infection with AIVs. Nearly half (31/65) of the H5N6 cases occurred in residents of rural areas, where birds were raised. Among the 65 cases, information regarding history of poultry exposure was available for 61 (93.8%) persons; the most common exposures were visiting a live poultry market (38, 62.3%) and exposure to backyard poultry (28, 45.9%). Two cases reported in August 2021 were in a husband and wife, both of whom reported poultry exposure. All close contacts were investigated, and no influenza-like illness symptoms were observed for any household contacts. However, human-to-human transmission cannot be ruled out for the infections in the husband and wife.

### Clinical Findings 

Of the 65 case-patients, 31 reported comorbidities (Table 1), 24 were otherwise healthy, and 10 did not report information on other health conditions. The prevalence of coronary heart disease and cancer in influenza H5N6 case-patients was higher than in the general population in China (Appendix Table 2).

The most commonly reported symptoms at onset of illness were fever (73.4%) and cough (59.4%) (Table 2), indicating that H5N6 infection is difficult to distinguish from other respiratory illnesses, including seasonal influenza viruses, in terms of early symptoms. The median onset-to-admission interval was 4 days (IQR 2–6 days), and the median onset-to-laboratory confirmation delay was 9 days (IQR 7–12 days).

**Table 2:** List of symptoms at illness onset of 64 laboratory-confirmed cases of human infection with avian influenza A(H5N6) virus, China*

**Symptoms at illness onset**	**No. (%)**
**Fever (>38°C)**	47 (73.4)
**Cough**	38 (59.4)
**Sputum**	15 (23.4)
**Fatigue**	15 (23.4)
**Dizziness**	14 (21.9)
**Chills**	14 (21.9)
**Headache**	13 (20.3)
**Shortness of breath**	9 (14.1)
**Muscle soreness**	7 (10.9)
**Sore throat**	6 (12.2)
**Nasal congestion**	6 (9.4)
**Coryza**	5 (7.8)
**Vomiting**	4 (6.3)
**Chest pain**	2 (3.1)

*Information for 1 case was not available.

**Figure 3:**
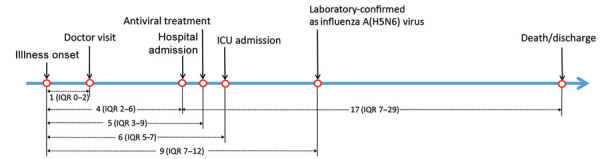
Disease courses of human infections with influenza A (H5N6) virus, China, April 21, 2014–December 31, 2021. Intervals are given as median (ICU) days. ICU, intensive care unit; IQR, interquartile range.

Antiviral treatments targeting NA were given to 60% of patients. However, the median time from illness onset to antiviral treatment was 5 days (IQR 3–9 days) (Figure 3). For patients in whom time from illness onset to initiation of antiviral treatment was <5 days, CFR was 38.1% (8/21), lower than in patients for whom that interval was >5 days (66.7% [12/18]). For case-patients who received no antiviral treatment, CFR was 65.2% (15/23). Although not statistically significant (p = 0.13), differences did exist in the CFR value among these groups, indicating that early initiation of antiviral treatments could increase the survival rate of H5N6 patients.

Severe disease occurred in 93.8% of cases, and complications developed in 86.2% of patients (Table 1). A total of 51 case-patients required admission to the intensive care unit for treatment; 41 persons underwent mechanical ventilation, and 14 persons required extracorporeal membrane oxygenation (Table 1). We estimated that the hospitalization fatality risks were 59.0% (95% CI 46.7%–71.4%). No significant difference was observed in terms of mean age between fatal cases (45.2 + 19.9 years) and survivors (45.1 + 20.2 years).

### Comparative Epidemiology of H5N6 Infections 

To identify any possible changes to risk for H5N6 infection before and during 2021 in China, we comparatively analyzed epidemiologic and clinical characteristics of human infections. We observed no significant difference in fatality rate, sex distribution, or the course of disease (Table 3; Appendix Figure 1). However, differences between the seasonal distributions of H5N6 infections were evident (Figure 1). In previous years, few cases were reported during June–September, but an increase in H5N6 infections occurred during summer 2021. In addition, we observed a marked difference in case-patients’ geographic location in 2021 (Table 3). Approximately 34.5% of H5N6 case-patients before 2021 lived in rural areas; during 2021, ≈58.3% of case-patients lived in rural areas (p<0.05). Furthermore, the average age of patients who were infected with or died of H5N6 viruses was much lower before 2021 than during 2021. The median age of those infected with H5N6 viruses was 40 years (IQR 25–50 years) before 2021 versus 54 years (IQR 49.5–60.5 years) during 2021 (p<0.001) (Table 3). The mean age of persons who died of H5N6 infection was 37.7 years (95% CI 27.9–47.4, SD 19.6 years) before 2021 versus 52.8 years (95% CI 44.0–61.6,; SD 17.7 years) during 2021 (p<0.05). Of note, third-party sequencing agencies started to play a role in diagnosing H5N6 viruses after the COVID-19 pandemic began. More than one third of cases in 2021 were first detected by hospitals sending samples from patients with pneumonia to third-party agencies.

**Table 3:** Comparison of characteristics of laboratory-confirmed cases of human infection with avian influenza A(H5N6) virus before and during 2021, China*

**Characteristic**	**No. case-patients before 2021, n = 29**	**No. case-patients during 2021, n = 36**	**p value**
**Sex**			
** M **	12 (41.4)	21 (58.3)	0.17
** F**	17 (58.6)	15 (41.7)	
**Median age, y (IQR)**	40 (25-50)	54 (49.5–60.5)	<0.001
**Age group, y**			<0.05
** 0–15**	6 (20.7)	2 (5.6)	
** 16–59**	21 (72.4)	24 (66.7)	
**>60**	2 (6.9)	10 (27.8)	
**Rural residence**	10 (34.5)	21 (58.3)	0.06
**Fatality**	18 (62.1)	18 (53.0)	0.47
Poultry exposure **<**10 d before illness onset			
** Any exposure to poultry**	25 (86.2)	36 (100.0)	<0.05
** Visited live poultry market**	18 (78.3)	20 (58.8)	0.13
** Exposure to backyard poultry**	8 (34.8)	20 (58.8)	0.07
** Exposure to sick or dead poultry**	7 (30.4)	16 (45.7)	0.24
** Processed poultry: slaughtered, cleaned, depilated, cooked**	11 (50.0)	17 (51.5)	0.91
**Comorbidities†**			
** Any‡**	11 (44.0)	20 (66.7)	0.09
** Hypertension**	3 (12.0)	11 (36.7)	<0.05
** Diabetes**	2 (8.0)	2 (6.7)	1.00
** Coronary heart disease**	3 (12.0)	5 (16.7)	0.72
** Cancer**	4 (16.0)	2 (6.7)	0.39
** Chronic renal disease**	0 (0.0)	4 (13.3)	0.12
**Median time from illness onset to hospital admission, d (IQR)**	5 (2.0–6.0)	4 (2.5–6.0)	0.61
**Median time from illness onset to laboratory confirmation, d (IQR)**	9 (7.0–12.0)	8.5 (7.0-12.0)	0.90
**Median time from illness onset to oseltamivir treatment, d (IQR)**	5 (3.0–9.0)	6 (2.0-8.0)	0.94
**Median time from illness onset to ICU admission, d (IQR)**	6 (5.0–7.0)	6 (4.5-8.0)	0.60
**Median time from hospital admission to death, d (IQR)**	16 (3.0–24.0)	12 (8.0–26.0)	0.50
**Mean time from hospital admission to discharge, d (SD)**	26.0 + 19.0	30.7 + 19.3	0.68

*Values are no. (%) except as indicated. IQR, interquartile range. †Only underlying diseases associated with a high risk for influenza complications (*32*) were counted here, including chronic respiratory disease, asthma, chronic cardiovascular disease, diabetes, chronic liver disease, chronic kidney disease, immunosuppressed status, and neuromuscular disorders. **‡Ten cases for which existence of comorbidities was unknown were excluded.**

### Evolutionary Relationships of HA and NA Genes of H5N6 Viruses Isolated from Humans 

**Figure 4:**
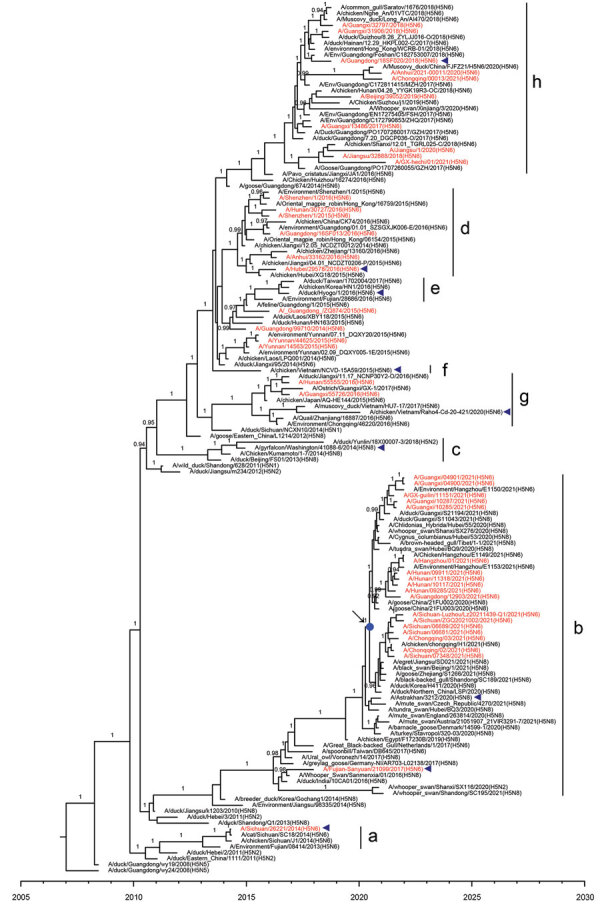
Maximum clade credibility trees of hemagglutinin gene of influenza A(H5N6) viruses, China. Red indicates human-origin H5N6 viruses; blue triangles indicate H5Ny vaccine strains recommended by the World Health Organization; blue dot indicates the most recent common ancestry of clade 2.3.4.4b A(H5N6) human viruses in 2021. Posterior probabilities >0.9 are labeled on the branches.

To elucidate the evolutionary pattern of divergence, we analyzed the genetic relationships of the HA and NA genes of 42 human H5N6 viruses with other clade 2.3.4.4 H5N6 genomes available in public databases. According to World Health Organization (WHO) nomenclature of HA genes in clade 2.3.4.4, HPAI H5N6 viruses can be divided into 8 subclades, a–h (Figure 4). All human isolates of H5N6 viruses clustered with vaccine strains recommended by the WHO. The first human virus isolated in 2014, A/Sichuan/26221/2014(H5N6), belonged to subclade 2.3.4.4a; 5 viruses isolated in 2015 were grouped into subclade 2.3.4.4d; and viruses isolated in 2016 belonged to subclade 2.3.4.4g. Subclade 2.3.4.4h viruses were isolated from 2015 through 2021. Of note, most H5N6 human viruses in 2021 (18/20) grouped into genetic subclade 2.3.4.4b. The median tMRCA among the HA genes of clade 2.3.4.4b H5N6 human viruses in 2021 was estimated to be June 16, 2020 (95% highest posterior density March 29, 2020–August 23, 2020).

**Figure 5:**
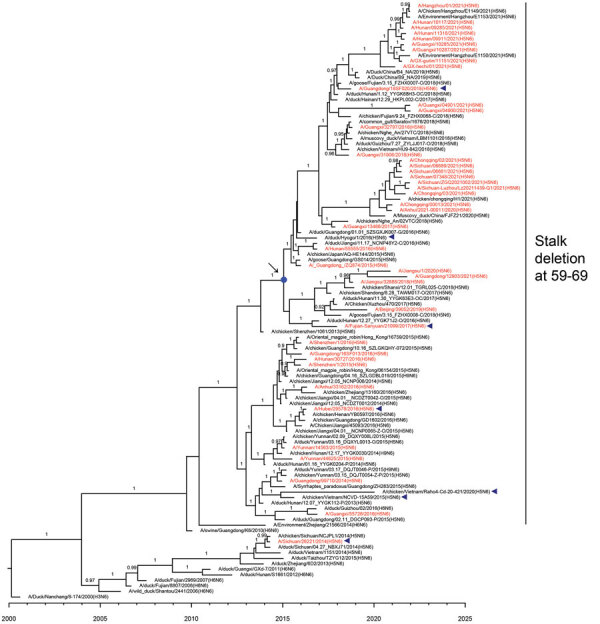
Maximum clade credibility tree of neuraminidase gene of influenza A(H5N6) viruses, China. Red indicates human-origin H5N6 viruses; blue triangles indicate vaccine strains recommended by the World Health Organization; blue dot indicates the most recent common ancestry of clade 2.3.4.4b H5N6 human viruses in 2021. Posterior probabilities >0.9 are labeled on the branches.

Phylogenetic analysis of the N6 genes revealed 2 subclades (Figure 5). Almost all viruses fell into the subgroup with an 11-residue deletion at position 59–69 in the NA stalk. Only 1 virus, A/Sichuan/26221/2014(H5N6), had full-length NA protein. The tMRCA among the N6 genes of clade 2.3.4.4b H5N6 human viruses from 2021 was estimated to be January 19, 2015 (95% highest posterior density June 16, 2014–June 17, 2015). These results indicated that clade 2.3.4.4b H5N6 viruses were generated by multiple reassortment events.

### Classification and Temporal Distribution of H5N6 Genotypes Isolated from Humans 

**Figure 6:**
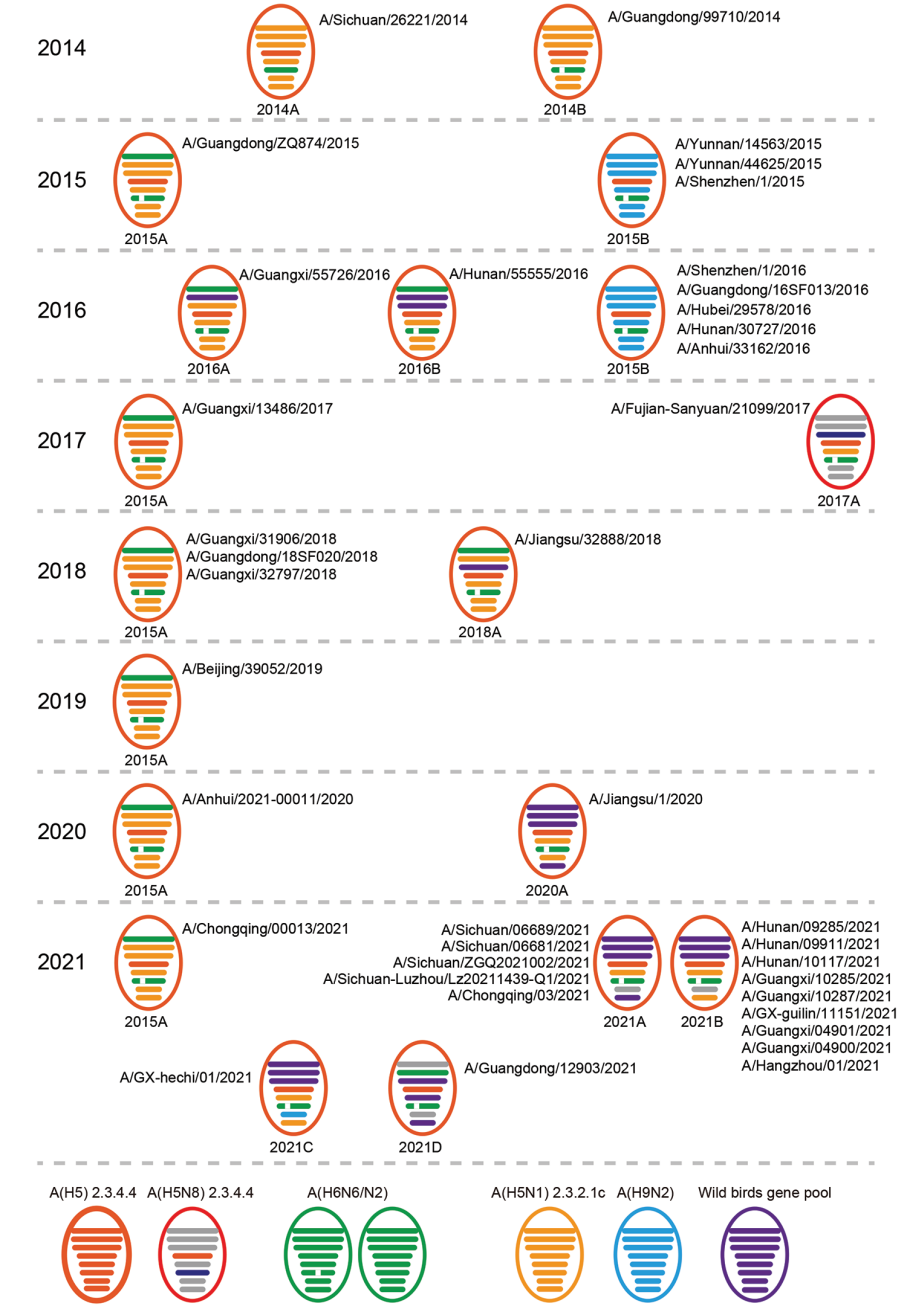
Diversity and prevalence of influenza A(H5N6) viruses isolated from humans, China. Circles represent the corresponding virus genotypes and their times of isolation. Gene segments are ordered as polymerase basic 2, polymerase basic 1, polymerase acidic, hemagglutinin, nucleoprotein, neuraminidase, matrix, and nonstructural from top to bottom within circles. A total of 13 genotypes are listed; the genotype name is shown under each circle. Names of each human H5N6 virus are listed besides the genotype to which they belong. To illustrate the history of reassortant events, segments in descendant viruses are colored according to their corresponding source viruses on the bottom line.

The phylogenetic analysis exhibited dynamic donation of the 8 gene segments to H5N6 viruses, including those from the Eurasian gene pool, H9N2 AIVs, and so on (Appendix Figure 2). Each gene of the human isolates showed a highly homologous sequence origin from poultry viruses, wild bird viruses, or both. On the basis of different clade combinations of 6 internal genes, we identified a total of 13 H5N6 genotypes, termed 2014A, 2014B, 2015A, 2015B, 2016A, 2016B, 2017A, 2018A, 2020A, 2021A, 2021B, 2021C, and 2021D (Figure 6). Several of these genotypes (e.g., 2014A and 2014B) were only identified in 1 case. In contrast, genotype 2015A was first detected in 2015 but continuously caused human infections during 2017–2021. Genotype 2015B contained a set of internal genes derived from H9N2 viruses in poultry and infected at least 8 persons during 2015–2016. However, no cases infected with this genotype were detected in the years after. Genotype 2020A emerged in 2020 and had 6 internal genes originating from the Eurasian gene pool and clade 2.3.2.1c H5N1 viruses. In 2021, a total of 4 new genotypes (2021A, 2021B, 2021C, and 2021D) acquiring genes from clade 2.3.4.4b H5N8 viruses, H5N1 viruses, and the Eurasian gene pool were identified. Five human isolates from Sichuan and Chongqing provinces belonged to genotype 2021A, and 9 human isolates from Hunan, Guangxi, and Zhejiang provinces were classified into genotype 2021B. Genotype 2021C and 2021D contained 1 virus each. Partial sequences were obtained from another 3 human viruses detected in 2021; their available sequences were closely related to those of the new genotype viruses. These findings might indicate the cross-species advantages of these newly emerged H5N6 genotypes to infect humans.

### Key Amino Acid Mutations Occurring in H5N6 Viruses Isolated from Humans

We analyzed molecular substitutions associated with increased virulence and transmissibility in mammals and reduced susceptibility to antiviral drugs. All H5N6 human viruses obtained a 5–amino acid insertion in the HA cleavage site, except for A/Anhui/33162/2016(H5N6), which had 1 more amino acid insertion. Substitution Q226L (H3 numbering) in HA protein, which had previously been reportedly associated with a switch in receptor specificity from avian-type (α2–3Gal) to human-type (α2–6Gal) (*34*–*36*), was detected from 2 viruses, A/ Sichuan-Luzhou/LZ20211439-Q1/2021(H5N6) and A/Hunan/09285/2021(H5N6). Substitution S227R in HA protein, which could also alter receptor specificity (*37*), was detected in 32 human viruses. Another receptor-changing substitution, T192I (*38*), was newly detected in clade 2.3.4.4b viruses from 2021 cases (Table 4; Appendix Table 3).

**Table 4:** Mammalian adaptation–related molecular markers of the human and nonhuman A(H5N6) viruses, China

Protein	Biologic effect	Mutations	Amino acids	Human viruses	Nonhuman viruses
HA*	Altered receptor specificity	T192I	T	22	1,238
			A	2	24
			I	18	36
			K	0	2
	Altered receptor specificity	Q226L	Q	39	1,302
			L	2	0
			Q\R	1	0
	Altered receptor specificity	S2227N/R	S	6	136
			G	3	31
			H	1	2
			H/R	2	0
			Q	0	92
			R	30	1,036
			C	0	4
	Altered receptor specificity	G228S	G	42	1,302
NA†	Reduced susceptibility to neuraminidase inhibitors	E119V/A/D	E	41	1,253
			D	1	34
			G	0	1
M2	Reduced susceptibility to amantadine	V27A	V	41	1,154
			A	1	20
			G	0	9
			I	0	15
	Reduced susceptibility to amantadine	A30V/T/S	A	41	1,197
			A/T	1	0
			S	0	1
	Reduced susceptibility to amantadine	S31N/G	S	33	994
			N	9	204
PA	Reduced susceptibility to endonuclease inhibitors	I38M/T/S/L	I	39	1,181
			L	0	1
			M	0	1
			V	1	1
PB2	Increased virulence in mammalian models	Q591K	Q	40	1,194
			K	1	0
	Increased virulence in mammalian models	E627K	E	28	1,180
			K	12	3
			E/V	1	1
			V	0	9
	Increased virulence in mammalian models	D701N	D	36	1,193
			D/N	1	0
			N	4	0
NS1	Altered virulence in mice	D92E	D	18	185
			E	24	1,011
			G	0	1
	Altered virulence in mice	L103F	L	8	165
			F	26	1,020
			S	0	11
			V	7	1
			Y	1	0
	Altered virulence in mice	I106M	I	8	166
			K	0	4
			M	34	1,027
M1	Altered virulence in mice	N30D	D	42	1,200
	Impacts growth and transmission in the guinea pig	P41A	A	42	1,199
			S	0	1
	Altered virulence in mice	T139A	T	40	1,113
			A	2	58
			P	0	29
	Altered virulence in mice	T215A	A	42	1,200

*H3 numbering system was used. †N2 numbering system was used.

Several mammalian-adapted mutations have occurred in PB2 protein of H5N6 viruses (Appendix Table 3). The substitutions E627K and D701N in PB2 protein, which were associated with increased polymerase activities or enhanced virulence in mice, occurred in 12/42 (E627K) and 5/42 (D701N) human viruses. A high proportion of these substitutions were observed in genotype 2015B viruses; 6/8 viruses acquired the E627K substitution and 1/8 viruses acquired the D701N substitution. These phenomena were frequently observed when an AIV transmitted to a mammalian host (*39*–*42*). These findings further confirmed that the infection source of H5N6 cases was poultry populations.

All genotype H5N6 viruses had 31S in M2 protein, except for genotype 2015B viruses. The genotype 2015B viruses contained 8 viruses, and exclusively had 31N in M2 protein, indicating their reduced susceptibility to amantadine (*43*). Mutations associated with drug resistance in the NA protein (E119D) only occurred in 1 human virus A/Anhui/33162/2016(H5N6). Mutation of I38V in PA, which might affect susceptibility to endonuclease inhibitors, was found in A/Jiangsu/32888/2018(H5N6). In addition, residues in the M1 and NS1 proteins of several H5N6 viruses showed changes that might alter virulence in mice (Appendix Table 3).

## Discussion

Among AIVs, viruses of subtypes H5 and H7 have been of particular concern because of their high rates of death. The hospitalization fatality risk for H5N6 infections (59.0%) was slightly lower than that for H5N1 infections (70.0%) but higher than that for H7N9 (35.0%) (*44*). In addition, the median age of patients with H5N6 virus (51 years) was older than the median age for patients with H5N1 virus (26 years) but younger than the age for patients with H7N9 virus (62 years) (*44*).

On the basis of epidemiologic investigations, 93.8% of influenza H5N6 case-patients were confirmed to have poultry exposure history. The contamination of live poultry markets and backyard birds, as well as the practice of processing poultry without personal protection, could be ongoing exposure sources for influenza A(H5N6) virus. While H5N6 viruses continue to circulate in poultry, human infections will undoubtedly continue.

WHO has recommended early antiviral therapy, ideally within 48 hours of symptom onset, for suspected or confirmed influenza patients (*45*). Our study found that early initiation of antiviral treatments could reduce the fatality rate in H5N6 patients to some extent. However, symptoms at the onset of influenza H5N6 infections were clinically similar to those of other respiratory pathogen infections. Thus, increased sensitivity of diagnostic systems is needed to improve case identification and initiate timely antiviral treatment.

During the COVID-19 pandemic, diagnostic capacity for respiratory illnesses among human health systems in China, including hospitals, Centers for Disease Control and Prevention at different levels, and third-party agencies, was increased. In our study, clinical samples from 15 H5N6 cases during the COVID-19 pandemic (2 in 2020 and 13 in 2021) were first identified by third party agencies. This additional diagnostic capacity contributed to the detection of H5N6 cases reported in 2021.

Genetic analysis in our study detected at least 13 types of reassortant H5N6 viruses in infected humans in China (Figure 6). Origins of internal genes were dramatically diversified, indicating the advanced genetic compatibility of H5N6 viruses with other AIVs. In 2021, a total of 4 new H5N6 genotype viruses emerged and accounted for almost all of the H5N6 human viruses based on the available full genome. Of note, different from previously isolated viruses, the HA gene of almost all H5N6 human viruses in 2021 belonged to genetic clade 2.3.4.4b and was closely related to that of the first H5N8 isolate, which caused infection in a patient in Russia (*46*). Additional mammal-adapted mutations, including Q226L and T192I in the HA protein, which could increase the viral affinity for human cells, were also detected (Appendix Table 3), indicating the viral adaptation process from birds to humans.

In summary, although we observed a rise in the number of influenza A(H5N6) infections in 2021, the disease course and CFR were comparable to previously detected H5N6 cases. Antiviral drugs remain effective if used early. However, new genotype viruses and mammal-adapted substitutions emerged. Moreover, reports from OFFLU (https://www.offlu.org) and WHO have documented that clade 2.3.4.4h and 2.3.4.4b H5N6 viruses and clade 2.3.4.4b H5N8 viruses have been detected in poultry and wild birds in China. Considering the continuous viral circulation in birds and incidence of human infection, more H5N6 variants and genotypes with further advantages in humans might emerge. Increased attention to such emerging viruses is vital for public health and pandemic preparedness.

Appendix Additional information about epidemiologic, clinical, and genetic characteristics of human infections with influenza A(H5N6) viruses, China.
