# Supplementary material for: Epidemiologic, Clinical, and Genetic Characteristics of Human Infections with Influenza A(H5N6) Viruses, China
Source: Emerg Infect Dis. 2022 Jul;28(7):1332–44. doi: 10.3201/eid2807.212482 (PMC9239879; doi:10.3201/eid2807.212482)
Supplement: Appendix — Additional information about epidemiologic, clinical, and genetic characteristics of human infections with influenza A(H5N6) viruses, China. [file 21-2482-Techapp-s1.pdf]

Article DOI: https://doi.org/10.3201/eid2807.212482

Epidemiologic, Clinical, and Genetic Characteristics of Human Infections with Influenza A(H5N6) Viruses, China

Appendix

Appendix Table 1. Viruses sequenced in 2021

GISAID Access ID	Virus Name	Subtype	Passage History	Location	Collection Date	Host Age	Host Sex
EPI_ISL_1081369	A/Chongqing/00013/2021	H5N6	C1	Chongqinq	2021–01–02	51	Male
EPI_ISL_6585606	A/Hunan/11318/2021	H5N6	Original	Hunan	2021–10–03	66	Male
EPI_ISL_6585605	A/Guangxi/10287/2021	H5N6	E1	Guangxi	2021–09–01	48	Female
EPI_ISL_6585604	A/Guangxi/10285/2021	H5N6	E1	Guangxi	2021–08–21	55	Male
EPI_ISL_5159431	A/Sichuan/07348/2021	H5N6	Original	Sichuan	2021–07–19	65	Female
EPI_ISL_5159426	A/Hunan/10117/2021	H5N6	Original	Hunan	2021–09–11	40	Female
EPI_ISL_4568644	A/Chonqqing/02/2021	H5N6	Original	Chongqing	2021–07–09	66	Male
EPI_ISL_4568643	A/Hunan/09911/2021	H5N6	E1	Hunan	2021–09–03	58	Female
EPI_ISL_4568642	A/Hunan/09285/2021	H5N6	E1	Hunan	2021–08–07	54	Male
EPI_ISL_4558108	A/Sichuan/06689/2021	H5N6	original	Sichuan	2021–07–05	55	Male
EPI_ISL_10792669	A/Guangxi/04901/2021	H5N6	E1	Guangxi	2021–12–25	53	Male
EPI_ISL_10792670	A/Guangxi/04900/2021	H5N6	E1	Guangxi	2021–12–31	28	Male
EPI_ISL_10792672	A/Guangdong/12903/2021	H5N6	E1	Guangdong	2021–10–27	52	Male

Appendix Table 2. The association on prevalence of underling diseases with influenza A(H5N6) infections*

Underlying diseases	Chinese population	H5N6 AIV cases, no. (%)
Underlying disease (≥18y)	——	30 (63.83)
Hypertension (≥18y)	27.50% (*1*)	14 (29.79)
Diabetes (≥18y)	11.90% (*2*)	4 (8.51)
Coronary heart disease (≥15y)	0.10% (*3*)	8 (17.02)
Cancer (≥18y)	0.29% (*1*)	6 (12.77)
Chronic renal disease (≥18y)	8.20% (*1*)	4 (8.51)
Chronic obstructive pulmonary disease (≥40y)	13.60% (*1*)	1 (2.56)

*Data on the underlying diseases were available for 47 cases of ≥18y and 39 cases of ≥40y. AIV, avian influenza virus.

Appendix Table 3. List of mammalian adaptation related molecular markers of the human A(H5N6) viruses in China

			HA*						NA†	M2			PA	PB2			NS1			M1			
No.	Strains	Genotype	Clade	192	226	227	228	Cleavage site	119	27	30	31	38	591	627	701	92	103	106	30	41	139	215
1	A/Sichuan/26221/2014	2014A	2.3.4.4a	T	Q	R	G	PLREKRRKR↓G	E	V	A	S	I	Q	E	N	E	F	M	D	A	T	A
2	A/Guangdong/99710/2014	2014B	2.3.4.4	T	Q	R	G	PLRERRRKR↓G	E	V	A	S	I	Q	K	D	E	F	M	D	A	T	A
3	A/_Guangdong_/ZQ874/2015	2015A	2.3.4.4	T	Q	R	G	PLRERRRKR↓G	E	V	A	S	I	Q	E	D	E	F	M	D	A	T	A
4	A/Guangxi/13486/2017	2015A	2.3.4.4h	T	Q	R	G	PLRERRRKR↓G	E	V	A	S	I	Q	K	D	E	F	M	D	A	T	A
5	A/Guangdong/18SF020/2018	2015A	2.3.4.4h	T	Q	R	G	PLRERRRKR↓G	E	V	A	S	I	Q	E\V	D	E	F	M	D	A	T	A
6	A/Guangxi/31906/2018	2015A	2.3.4.4h	A	Q	R	G	PLRERRRKR↓G	E	V	A	S	I	Q	K	D	E	F	M	D	A	T	A
7	A/Guangxi/32797/2018	2015A	2.3.4.4h	A	Q	H	G	PLRERRRKR↓G	E	V	A	S	I	Q	K	D	E	F	M	D	A	T	A
8	A/Beijing/39052/2019	2015A	2.3.4.4h	T	Q	R	G	PPRERRRKR↓G	E	V	A	S	I	Q	E	D	E	F	M	D	A	T	A
9	A/Anhui/2021–00011/2020	2015A	2.3.4.4h	T	Q	R	G	PLRERRRKR↓G	E	V	A	S	I	Q	E	D	E	F	M	D	A	T	A
10	A/Chongqing/00013/2021	2015A	2.3.4.4h	T	Q	R	G	PLRERRRKR↓G	E	V	A	S	I	Q	E	D	E	F	M	D	A	T	A
11	A/Shenzhen/1/2015	2015B	2.3.4.4d	T	Q	S	G	PLRERRRKR↓G	E	V	A	N	I	Q	E	D	D	L	I	D	A	T	A
12	A/Yunnan/14563/2015	2015B	2.3.4.4	T	Q	R	G	PLRERRRKR↓G	E	V	A	N	I	Q	K	D	D	L	I	D	A	A	A
13	A/Yunnan/44625/2015	2015B	2.3.4.4	T	Q	R	G	PLRERRRKR↓G	E	V	A	N	I	Q	K	D	D	L	I	D	A	A	A
14	A/Shenzhen/1/2016	2015B	2.3.4.4d	T	Q	S	G	PLRERRRKR↓G	E	V	A	N	I	Q	K	D	D	L	I	D	A	T	A
15	A/Guangdong/16SF013/2016	2015B	2.3.4.4d	T	Q	S	G	PLRERRRKR↓G	E	V	A	N	I	Q	K	D	D	L	I	D	A	T	A
16	A/Anhui/33162/2016	2015B	2.3.4.4d	T	Q	S	G	PLRERRRKKR↓G	D	V	A	N	I	Q	K	D	D	L	I	D	A	T	A
17	A/Hubei/29578/2016	2015B	2.3.4.4d	T	Q	S	G	PLRERRRKR↓G	E	V	A	N	I	Q	E	D	D	L	I	D	A	T	A
18	A/Hunan/30727/2016	2015B	2.3.4.4d	T	Q	S	G	PLRERRRKR↓G	E	V	A	N	I	Q	K	N	D	L	I	D	A	T	A
19	A/Guangxi/55726/2016	2016A	2.3.4.4g	T	Q	R	G	PLRERRRKR↓G	E	V	A	S	I	Q	E	D	E	F	M	D	A	T	A
20	A/Hunan/55555/2016	2016B	2.3.4.4g	T	Q	R	G	PLRERRRKR↓G	E	A	A	S	I	Q	E	D	E	F	M	D	A	T	A
21	A/Fujian-Sanyuan/21099/2017	2017A	2.3.4.4b	T	Q	R	G	PLREKRRKR↓G	E	V	A	S	I	Q	E	D	D	F	M	D	A	T	A
22	A/Jiangsu/32888/2018	2018A	2.3.4.4h	T	Q	G	G	PLRERRRKR↓G	E	V	A	S	V	Q	E	D	E	F	M	D	A	T	A
23	A/Jiangsu/1/2020	2020A	2.3.4.4h	T	Q	G	G	PLRERRRKR↓G	E	V	A\T	S	I	Q	E	D	D	Y	M	D	A	T	A
24	A/Sichuan/06681/2021	2021A	2.3.4.4b	I	Q	R	G	PLREKRRKR↓G	E	V	A	S	I	Q	E	D	D	V	M	D	A	T	A
25	A/Sichuan/06689/2021	2021A	2.3.4.4b	I	Q	R	G	PLREKRRKR↓G	E	V	A	S	I	Q	E	D	D	V	M	D	A	T	A

			HA*						NA†	M2		PA	PB2			NS1			M1			
No.	Strains	Genotype	Clade	192	226	227	228	Cleavage site	119	27	31	38	591	627	701	92	103	106	30	41	139	215
26	A/Sichuan/ZGQ2021002/2021	2021A	2.3.4.4b	I	Q	R	G	PLREKRRKR↓G	E	V	A	S	I	Q	K	D	D	V	M	D	A	T	A
27	A/Sichuan-Luzhou/Lz20211439-Q1/2021	2021A	2.3.4.4b	I	L	R	G	PLREKRRKR↓G	E	V	A	S	I	Q	E	D	D	V	M	D	A	T	A
28	A/Chongqing/03/2021	2021A	2.3.4.4b	I	Q	R	G	LLREKRRKR↓G	E	V	A	S	I	Q	E	N	D	V	M	D	A	T	A
29	A/Guangxi/10285/2021	2021B	2.3.4.4b	I	Q	R	G	PLREKRRKR↓G	E	V	A	S	I	Q	E	D\N	E	F	M	D	A	T	A
30	A/Guangxi/10287/2021	2021B	2.3.4.4b	I	Q	R	G	PLREKRRKR↓G	E	V	A	S	I	Q	E	D	E	F	M	D	A	T	A
31	A/Guangxi/04900/2021	2021B	2.3.4.4b	I	Q	H\R	G	PLREKRRKR↓G	E	V	A	S	I	Q	E	D	E	F	M	D	A	T	A
32	A/Guangxi/04901/2021	2021B	2.3.4.4b	I	Q	R	G	PLREKRRKR↓G	E	V	A	S	I	Q	E	D	E	F	M	D	A	T	A
33	A/GX-guilin/11151/2021	2021B	2.3.4.4b	I	Q	R	G	PLREKRRKR↓G	E	V	A	S	I	Q	E	D	E	F	M	D	A	T	A
34	A/Hunan/09285/2021	2021B	2.3.4.4b	I	L	R	G	PLREKRRKR↓G	E	V	A	S	I	Q	E	D	E	F	M	D	A	T	A
35	A/Hunan/09911/2021	2021B	2.3.4.4b	I	Q	H\R	G	PLREKRRKR↓G	E	V	A	S	I	Q	E	D	E	F	M	D	A	T	A
36	A/Hunan/10117/2021	2021B	2.3.4.4b	I	Q	R	G	PLREKRRKR↓G	E	V	A	S	I	Q	E	D	E	F	M	D	A	T	A
37	A/Hangzhou/01/2021	2021B	2.3.4.4b	I	Q	R	G	PLREKRRKR↓G	E	V	A	S	I	Q	E	D	E	F	M	D	A	T	A
38	A/GX-hechi/01/2021	2021C	2.3.4.4h	T	Q	G	G	PLRERRRKR↓G	E	V	A	N	I	Q	E	D	E	F	M	D	A	T	A
39	A/Guangdong/12903/2021	2021D	2.3.4.4b	I	Q	R	G	PLREKRRKR↓G	E	V	A	S	I	Q	E	N	D	F	M	D	A	T	A
40	A/Chonqqing/02/2021	N/A	2.3.4.4b	I	Q\R	R	G	PLREKRRKR↓G	E	V	A	S	N/A	N/A	N/A	N/A	D	V	M	D	A	T	A
41	A/Hunan/11318/2021	N/A	2.3.4.4b	I	Q	R	G	PLREKRRKR↓G	E	V	A	S	I	K	E	D	E	F	M	D	A	T	A
42	A/Sichuan/07348/2021	N/A	2.3.4.4b	I	Q	R	G	PLREKRRKR↓G	E	V	A	S	N/A	Q	K	D	D	V	M	D	A	T	A

*H3 numbering system was used. N/A, not available.

†N2 numbering system was used.

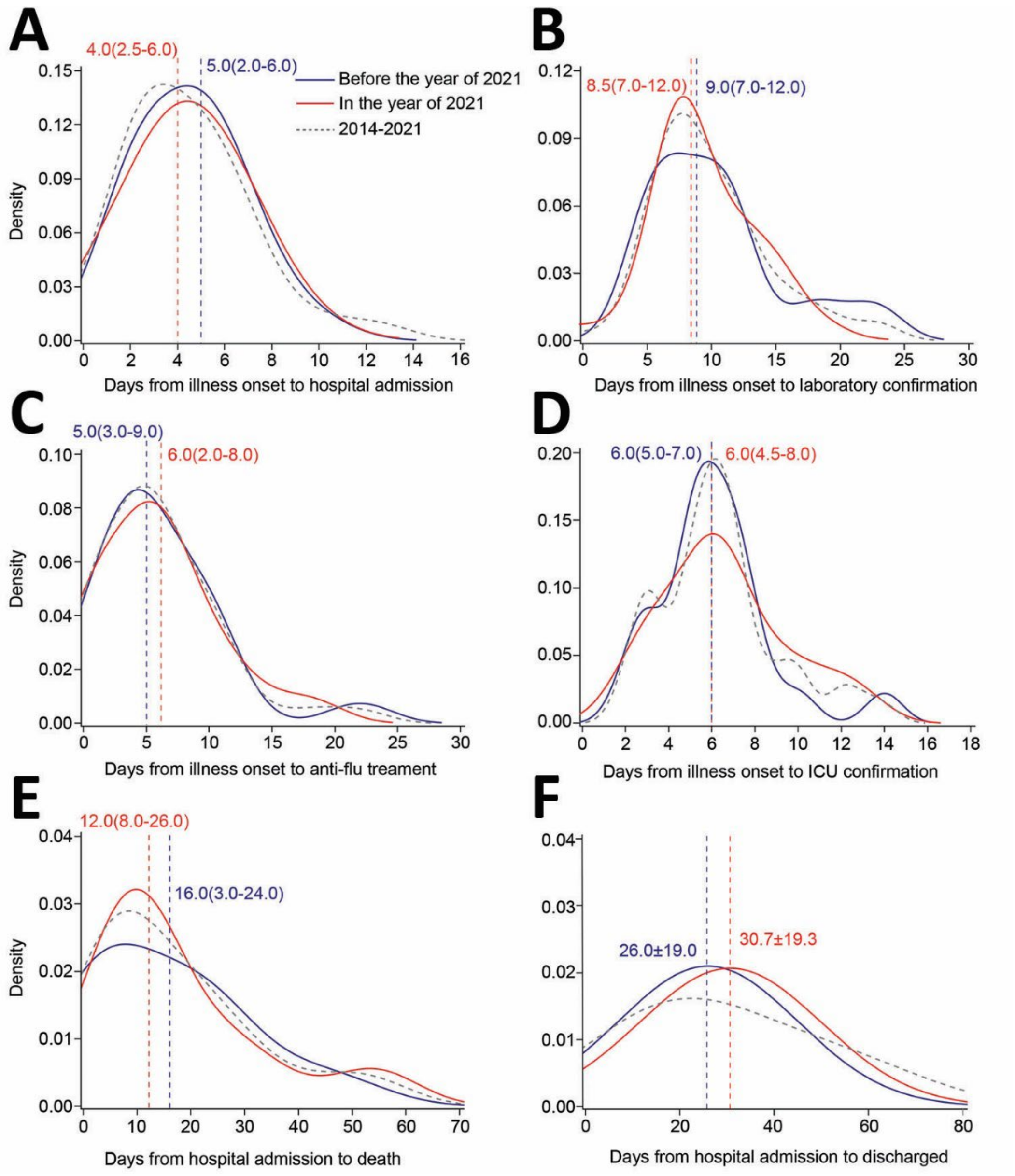

Appendix Figure 1. Time-delay distributions for laboratory-confirmed human infections with A(H5N6) influenza, China, 2014–2021. A) Days from illness onset to hospital admission, B) days from illness onset to laboratory confirmation of influenza A(H5N6) virus infection, C) days from illness onset to anti-influenza treatment, D) days from illness onset to ICU admission, E) days from hospital admission to death, F) days from hospital admission to discharged. The average days of delay are shown as dashed lines. Lines colored in blue and red represent time-delay distributions for A(H5N6) cases before and during 2021, respectively. Dashed gray line indicates the overall time-delay distributions for A(H5N6) infections during 2014–2021.

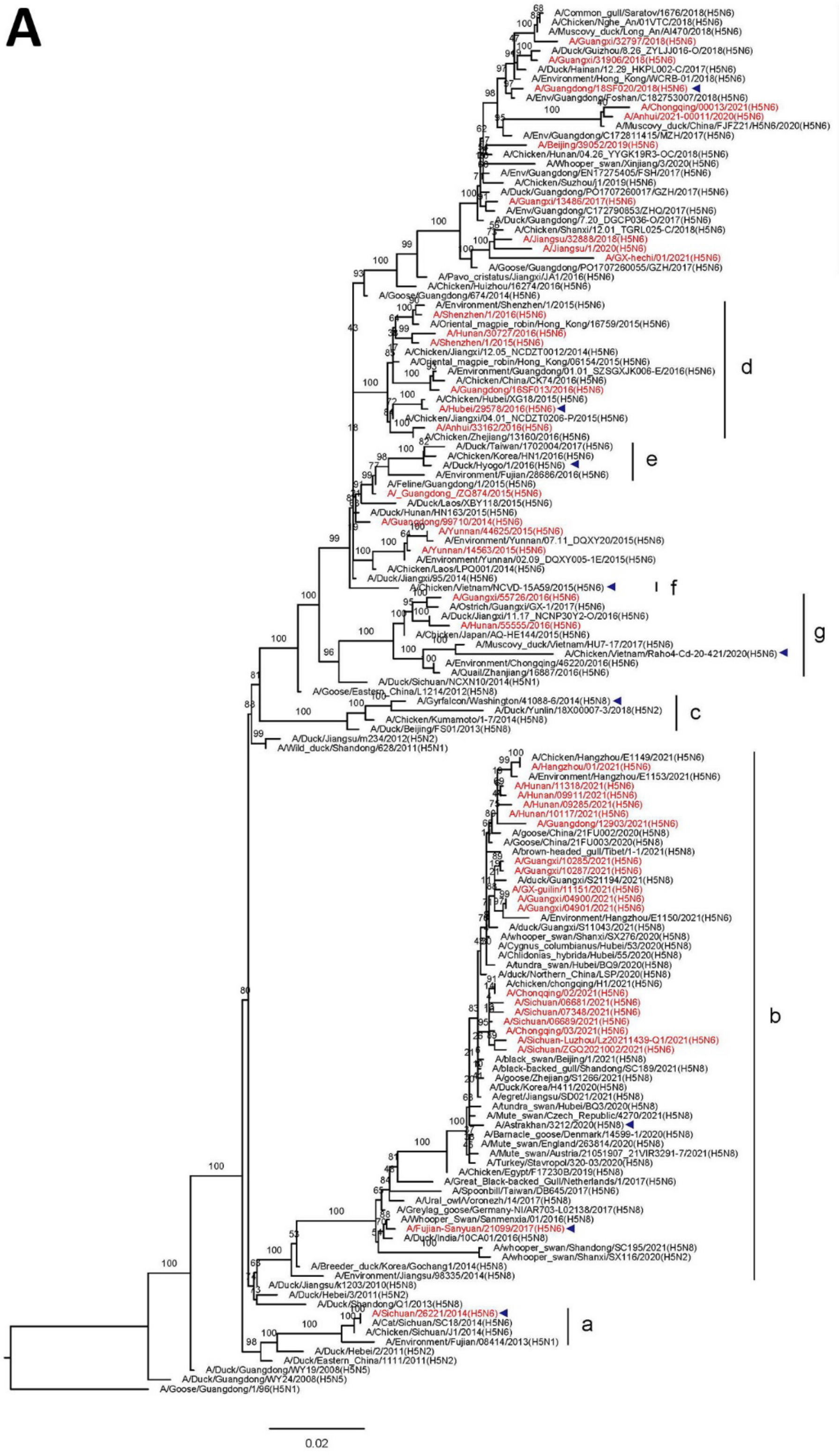
A
h
d
e
f
g
c
b
a
0.02

B

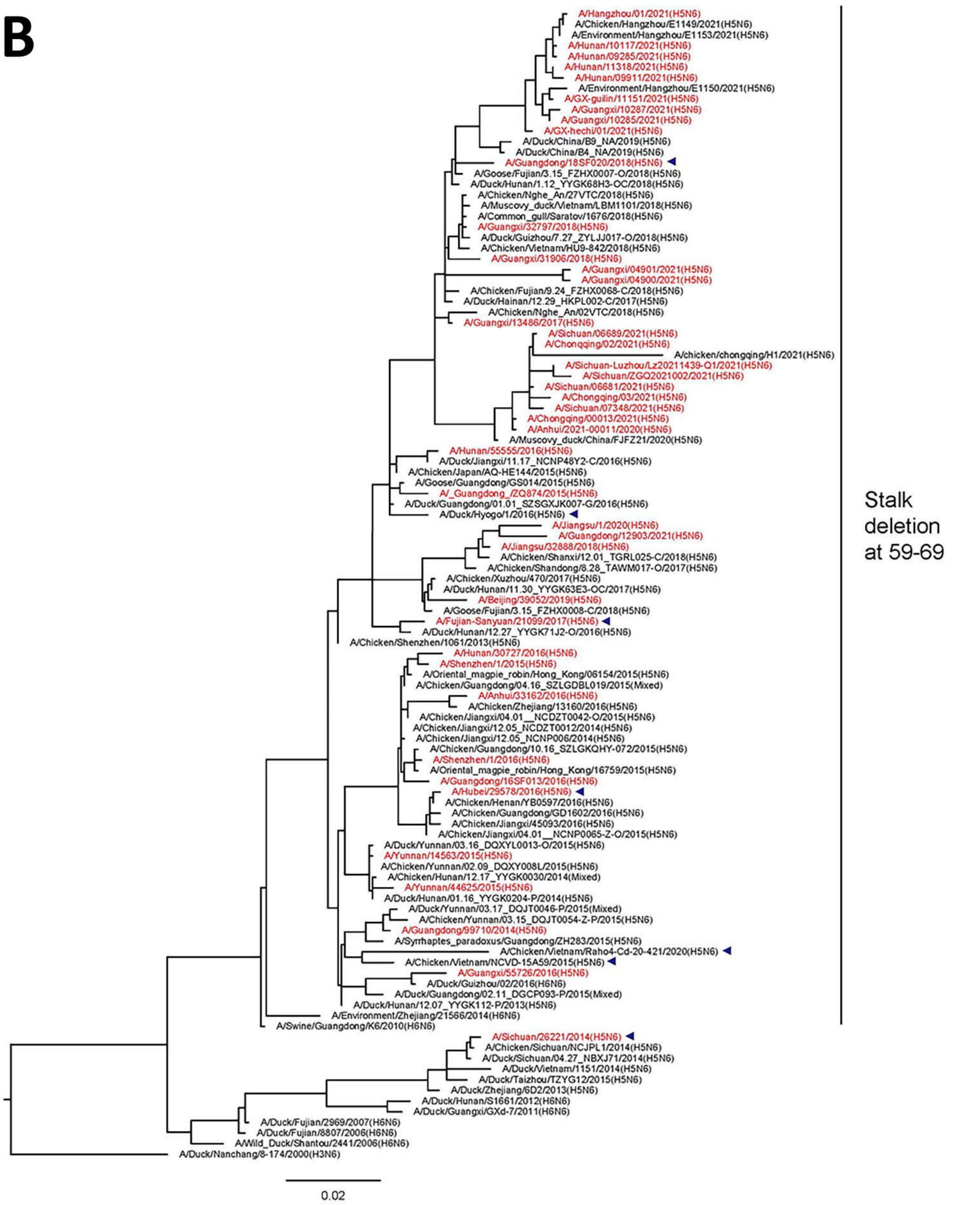

A/Hangzhou/01/2021(H5N6)
A/Chicken/Hangzhou/E1149/2021(H5N6)
A/Environment/Hangzhou/E1153/2021(H5N6)
A/Hunan/10117/2021(H5N6)
A/Hunan/09285/2021(H5N6)
A/Hunan/11318/2021(H5N6)
A/Hunan/09911/2021(H5N6)
A/Environment/Hangzhou/E1150/2021(H5N6)
A/GX-guilin/11151/2021(H5N6)
A/Guangxi/10287/2021(H5N6)
A/Guangxi/10285/2021(H5N6)
A/GX-hechi/01/2021(H5N6)
A/Duck/China/B9_NA/2019(H5N6)
A/Duck/China/B4_NA/2019(H5N6)
A/Guangdong/18SF020/2018(H5N6)
A/Goose/Fujian/3.15_FZHX0007-O/2018(H5N6)
A/Duck/Hunan/1.12_YYGK68H3-OC/2018(H5N6)
A/Chicken/Nghe_An/27VTC/2018(H5N6)
A/Muscovy_duck/Vietnam/LBM1101/2018(H5N6)
A/Common_gull/Saratov/1676/2018(H5N6)
A/Guangxi/32797/2018(H5N6)
A/Duck/Guizhou/7.27_ZYLJJ017-O/2018(H5N6)
A/Chicken/Vietnam/HU9-842/2018(H5N6)
A/Guangxi/31906/2018(H5N6)
A/Guangxi/04901/2021(H5N6)
A/Guangxi/04900/2021(H5N6)
A/Chicken/Fujian/9.24_FZHX0068-C/2018(H5N6)
A/Duck/Hainan/12.29_HKPL002-C/2017(H5N6)
A/Chicken/Nghe_An/02VTC/2018(H5N6)
A/Guangxi/13486/2017(H5N6)
A/Sichuan/06689/2021(H5N6)
A/Chonqqing/02/2021(H5N6)
A/chicken/chongqing/H1/2021(H5N6)
A/Sichuan-Luzhou/Lz20211439-Q1/2021(H5N6)
A/Sichuan/ZGQ2021002/2021(H5N6)
A/Sichuan/06681/2021(H5N6)
A/Chongqing/03/2021(H5N6)
A/Sichuan/07348/2021(H5N6)
A/Chongqing/00013/2021(H5N6)
A/Anhui/2021-00011/2020(H5N6)
A/Muscovy_duck/China/FJFZ21/2020(H5N6)
A/Hunan/55555/2016(H5N6)
A/Duck/Jiangxi/11.17_NCNP48Y2-C/2016(H5N6)
A/Chicken/Japan/AQ-HE144/2015(H5N6)
A/Goose/Guangdong/GS014/2015(H5N6)
A/_Guangdong_/ZQ874/2015(H5N6)
A/Duck/Guangdong/01.01_SZSGXJK007-G/2016(H5N6)
A/Duck/Hyogo/1/2016(H5N6)
A/Jiangsu/1/2020(H5N6)
A/Guangdong/12903/2021(H5N6)
A/Jiangsu/32888/2018(H5N6)
A/Chicken/Shanxi/12.01_TGRL025-C/2018(H5N6)
A/Chicken/Shandong/8.28_TAWM017-O/2017(H5N6)
A/Chicken/Xuzhou/470/2017(H5N6)
A/Duck/Hunan/11.30_YYGK63E3-OC/2017(H5N6)
A/Beijing/39052/2019(H5N6)
A/Goose/Fujian/3.15_FZHX0008-C/2018(H5N6)
A/Fujian-Sanyuan/21099/2017(H5N6)
A/Duck/Hunan/12.27_YYGK71J2-O/2016(H5N6)
A/Chicken/Shenzhen/1061/2013(H5N6)
A/Hunan/30727/2016(H5N6)
A/Shenzhen/1/2015(H5N6)
A/Oriental_magpie_robin/Hong_Kong/06154/2015(H5N6)
A/Chicken/Guangdong/04.16_SZLGDBL019/2015(Mixed)
A/Anhui/33162/2016(H5N6)
A/Chicken/Zhejiang/13160/2016(H5N6)
A/Chicken/Jiangxi/04.01__NCDZT0042-O/2015(H5N6)
A/Chicken/Jiangxi/12.05_NCDZT0012/2014(H5N6)
A/Chicken/Jiangxi/12.05_NCNP006/2014(H5N6)
A/Chicken/Guangdong/10.16_SZLGKQHY-072/2015(H5N6)
A/Shenzhen/1/2016(H5N6)
A/Oriental_magpie_robin/Hong_Kong/16759/2015(H5N6)
A/Guangdong/16SF013/2016(H5N6)
A/Hubei/29578/2016(H5N6)
A/Chicken/Henan/YB0597/2016(H5N6)
A/Chicken/Guangdong/GD1602/2016(H5N6)
A/Chicken/Jiangxi/45093/2016(H5N6)
A/Chicken/Jiangxi/04.01__NCNP0065-Z-O/2015(H5N6)
A/Duck/Yunnan/03.16_DQXYL0013-O/2015(H5N6)
A/Yunnan/14563/2015(H5N6)
A/Chicken/Yunnan/02.09_DQXY008L/2015(H5N6)
A/Chicken/Hunan/12.17_YYGK0030/2014(Mixed)
A/Yunnan/44625/2015(H5N6)
A/Duck/Hunan/01.16_YYGK0204-P/2014(H5N6)
A/Duck/Yunnan/03.17_DQJT0046-P/2015(Mixed)
A/Chicken/Yunnan/03.15_DQJT0054-Z-P/2015(H5N6)
A/Guangdong/99710/2014(H5N6)
A/Syrrhaptes_paradoxus/Guangdong/ZH283/2015(H5N6)
A/Chicken/Vietnam/Raho4-Cd-20-421/2020(H5N6)
A/Chicken/Vietnam/NCVD-15A59/2015(H5N6)
A/Guangxi/55726/2016(H5N6)
A/Duck/Guizhou/02/2016(H6N6)
A/Duck/Guangdong/02.11_DGCP093-P/2015(Mixed)
A/Duck/Hunan/12.07_YYGK112-P/2013(H5N6)
A/Environment/Zhejiang/21566/2014(H6N6)
A/Swine/Guangdong/K6/2010(H6N6)
A/Sichuan/26221/2014(H5N6)
A/Chicken/Sichuan/NCJPL1/2014(H5N6)
A/Duck/Sichuan/04.27_NBXJ71/2014(H5N6)
A/Duck/Vietnam/1151/2014(H5N6)
A/Duck/Taizhou/TZYG12/2015(H5N6)
A/Duck/Zhejiang/6D2/2013(H5N6)
A/Duck/Hunan/S1661/2012(H6N6)
A/Duck/Guangxi/GXd-7/2011(H6N6)
A/Duck/Fujian/2969/2007(H6N6)
A/Duck/Fujian/8807/2006(H6N6)
A/Wild_Duck/Shantou/2441/2006(H6N6)
A/Duck/Nanchang/8-174/2000(H3N6)
0.02
Stalk deletion at 59-69

C

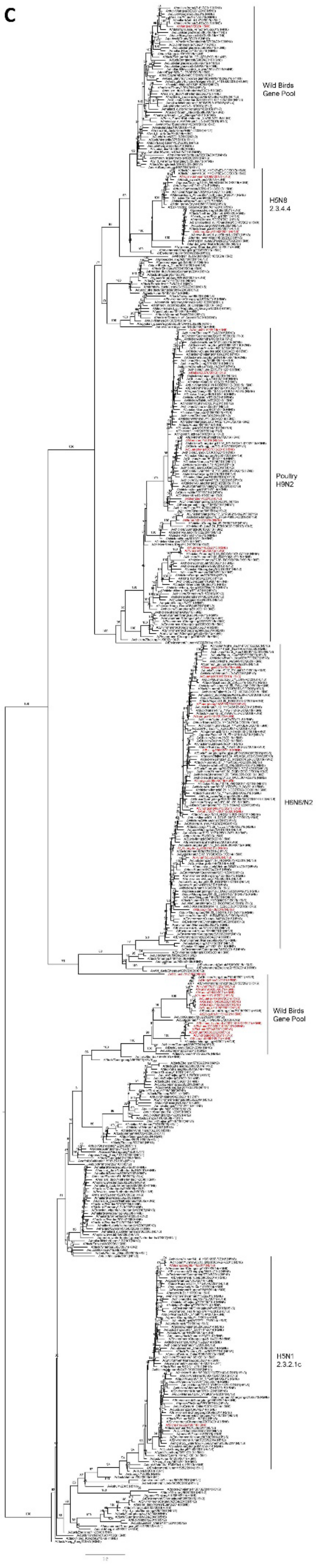
Wild Birds
Gene Pool
H5N8
2.3.4.4
Poultry
H9N2
H5N8/N2
Wild Birds
Gene Pool
H5N1
2.3.2.1c

D

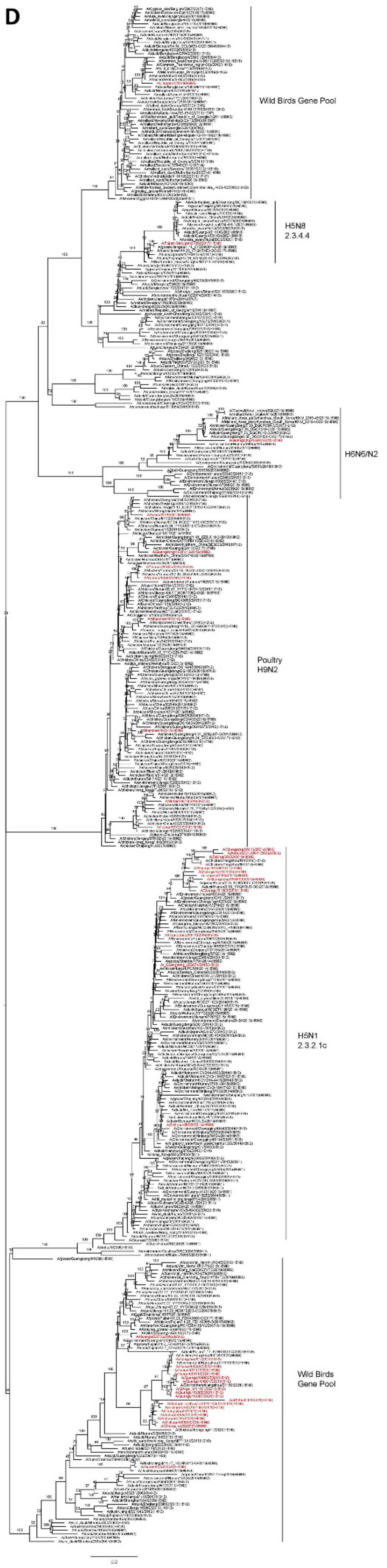

Wild Birds Gene Pool
H5N8
2.3.4.4
H6N6/N2
Poultry
H9N2
H5N1
2.3.2.1c
Wild Birds
Gene Pool
0.2

E

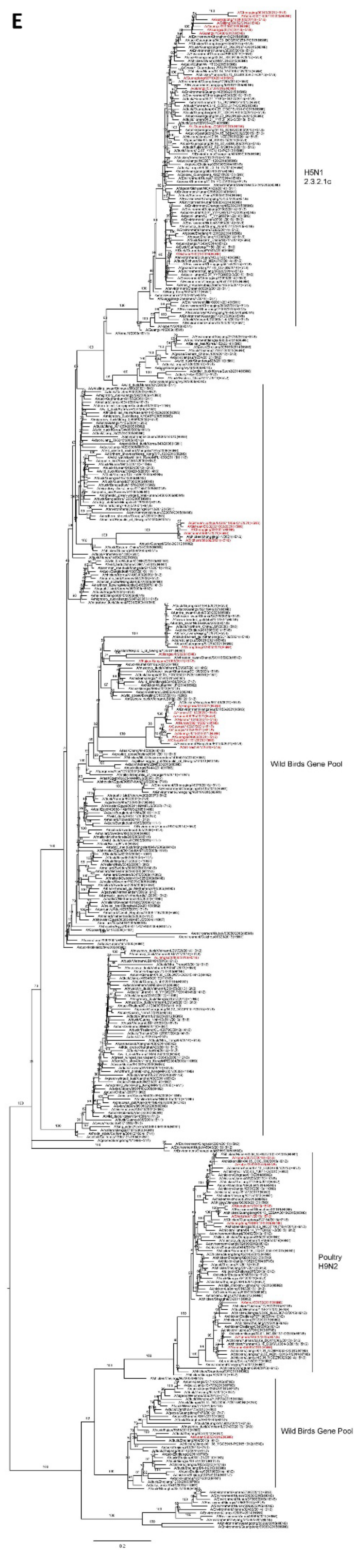
H5N1
2.3.2.1c
Wild Birds Gene Pool
Poultry
H9N2
Wild Birds Gene Pool
0.2

F

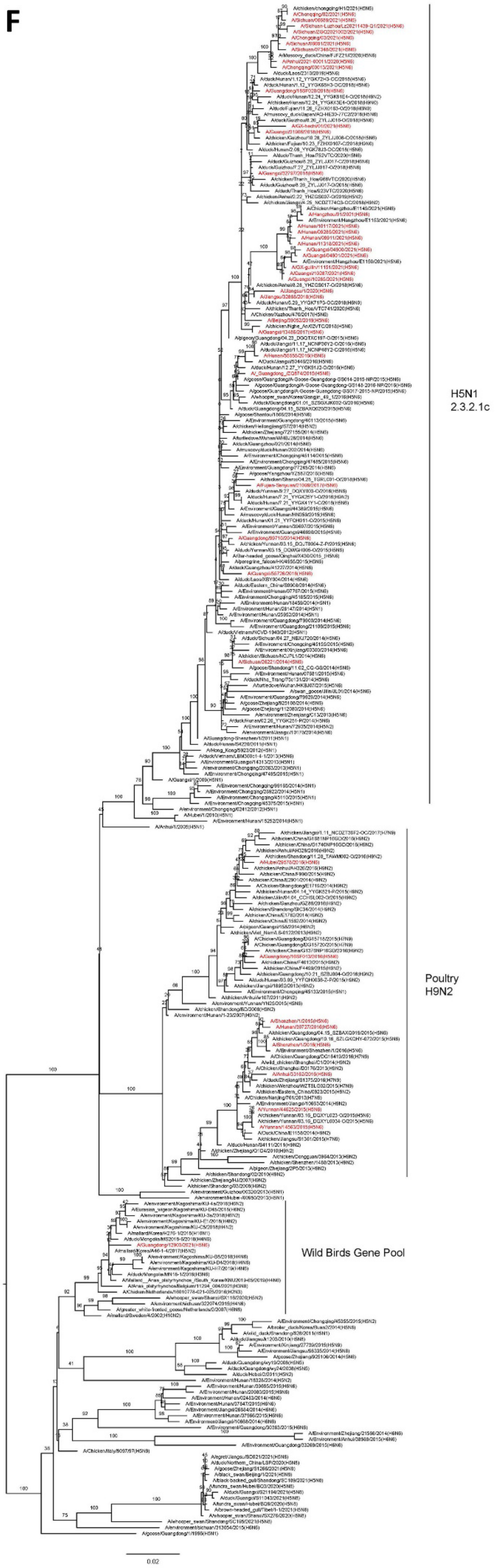

G

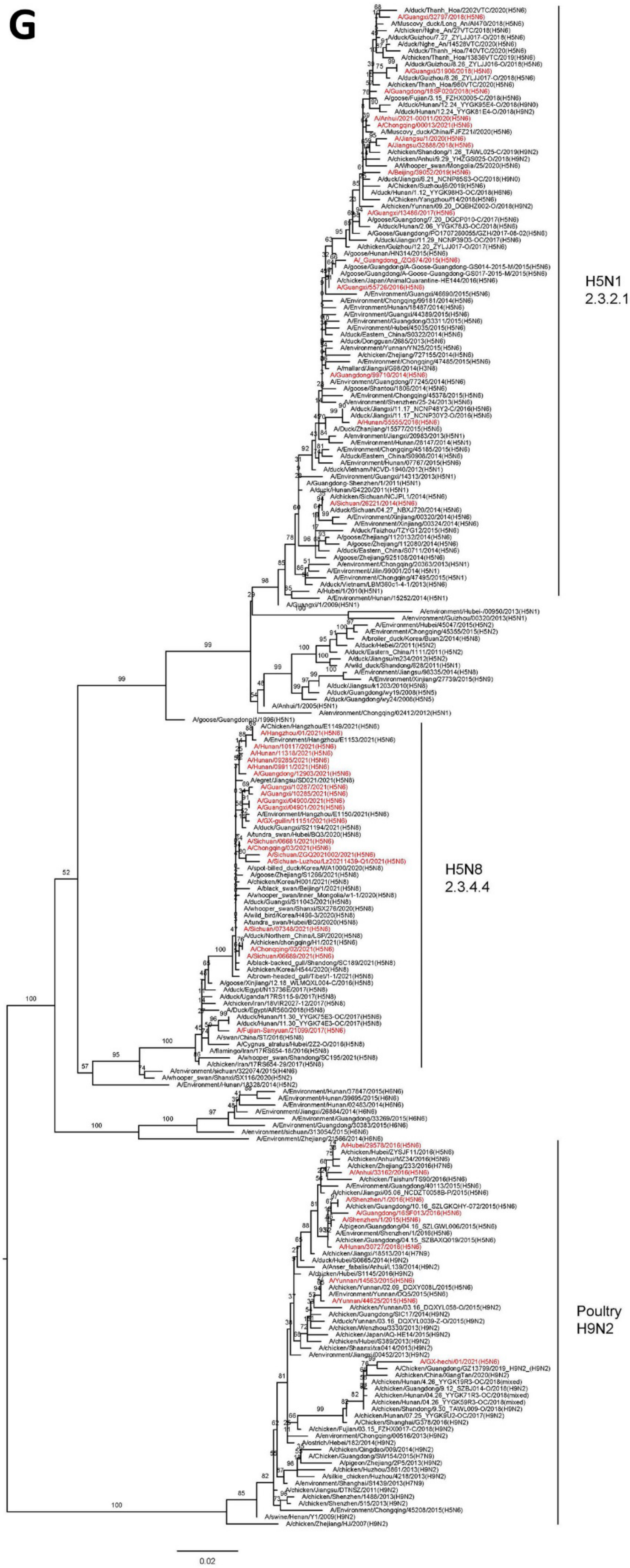

H

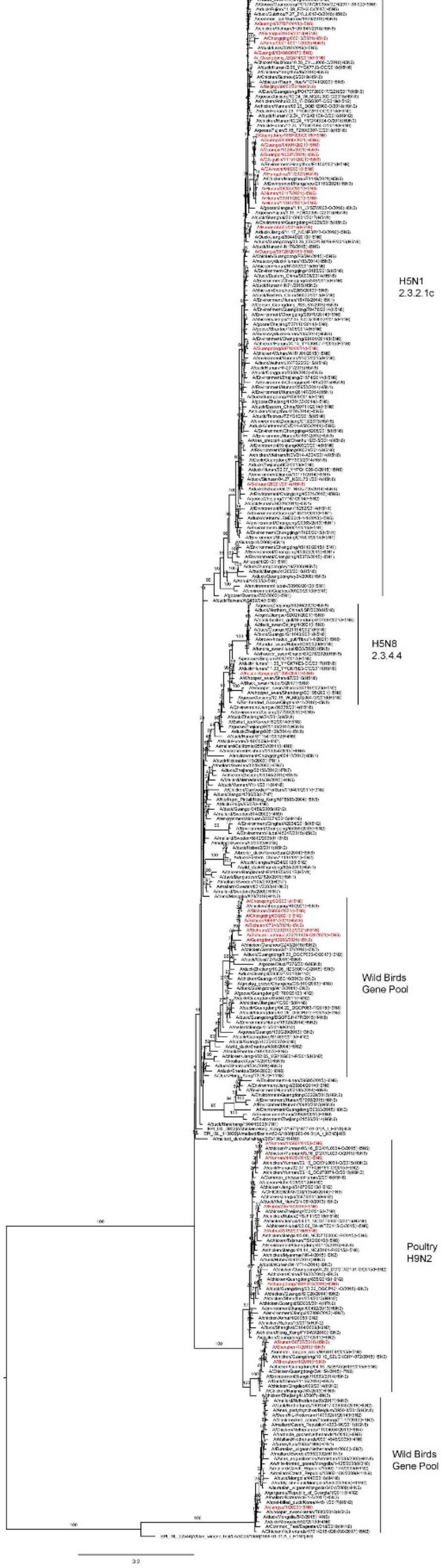

Appendix Figure 2. Evolution of the eight genes of influenza A (H5N6) viruses.Phylogenetic trees of HA, NA, PB2, PB1, PA, NP, MP, and NS genes were shown in A, B, C, D, E, F, G and H, respectively. Human origin A(H5N6) viruses were colored in red.

References

1. National Health Commission of the People's Republic of China. Report on Chinese residents' chronic diseases and nutrition 2020. Beijing: People's Medical Publishing House; 2020.

2. Chinese Center for Disease Control and Prevention. Report on chronic disease risk factor surveillance in China 2018. Beijing: People's Medical Publishing House; 2021.

3. The Writing Committee of the Annual Report on Cardiovascular Health and Diseases in China. Annual report on cardiovascular health and diseases in China 2020. Journal of Cardiovascular and Pulmonary Diseases. 2021;40:885–9.
